# Supplementary material for: High-Throughput De Novo Protein Design Yields Novel Immunomodulatory Agonists
Source: bioRxiv. 2025 Oct 13:2025.10.12.681920. Preprint. [Version 1] doi: 10.1101/2025.10.12.681920 (PMC12632978; doi:10.1101/2025.10.12.681920)
Supplement: 1 [file NIHPP2025.10.12.681920v1-supplement-1.pdf]

bioRxiv preprint doi: https://doi.org/10.1101/2025.10.12.681920; this version posted October 13, 2025. The copyright holder for this preprint (which was not certified by peer review) is the author/funder, who has granted bioRxiv a license to display the preprint in perpetuity. It is made available under aCC-BY 4.0 International license.

Supplementary Figures and Tables

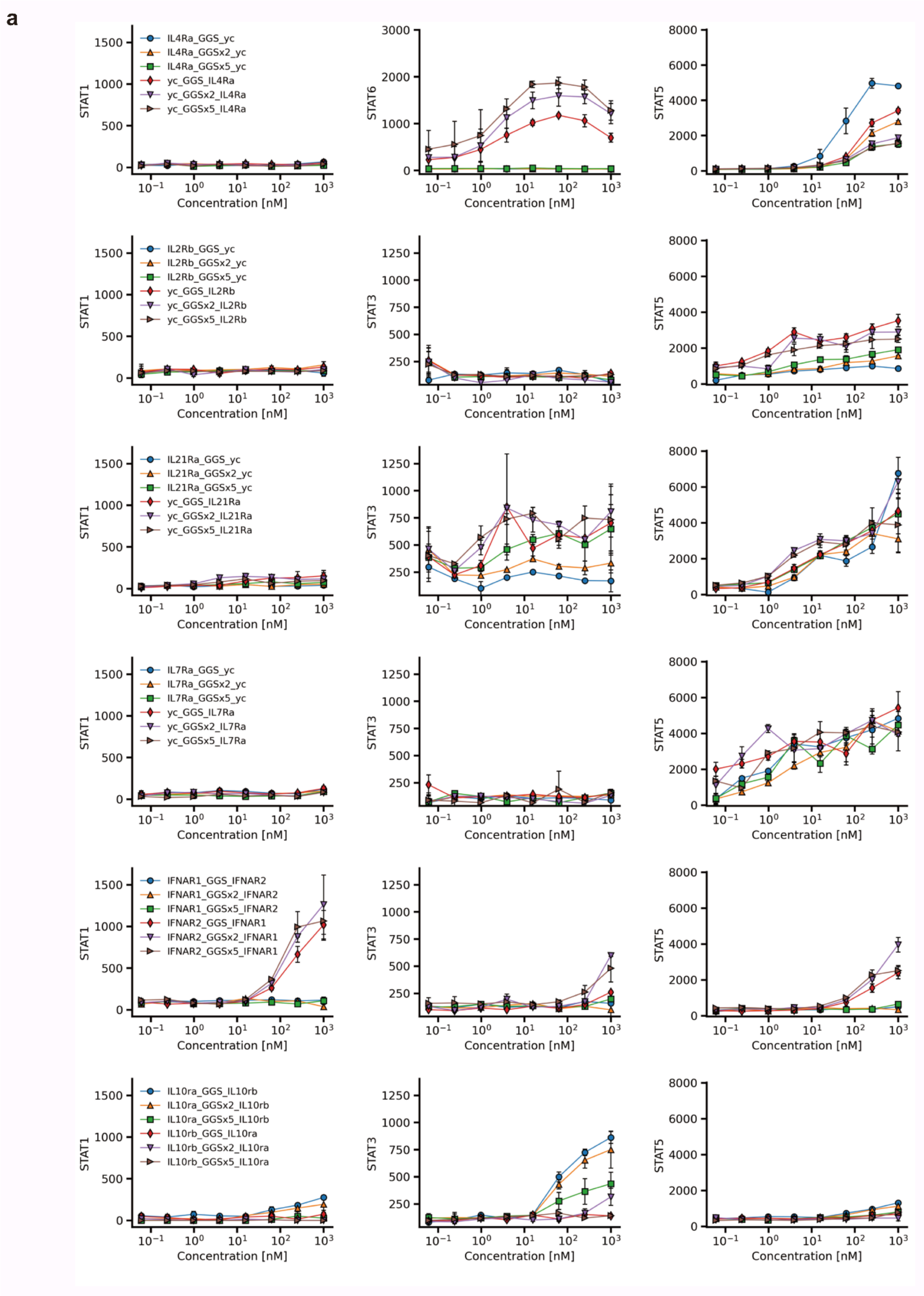

bioRxiv preprint doi: https://doi.org/10.1101/2025.10.12.681920; this version posted October 13, 2025. The copyright holder for this preprint (which was not certified by peer review) is the author/funder, who has granted bioRxiv a license to display the preprint in perpetuity. It is made available under aCC-BY 4.0 International license.

Supplementary Figure 1: pSTAT signaling of novokine mimics of natural cytokines. (a) dose-response curves for pSTAT1, pSTAT3, pSTAT5, and pSTAT6 signaling that was used to generate the grid plot shown in Figure 2D. Ligands were tested in PBMCs that were stimulated for 15 minutes.

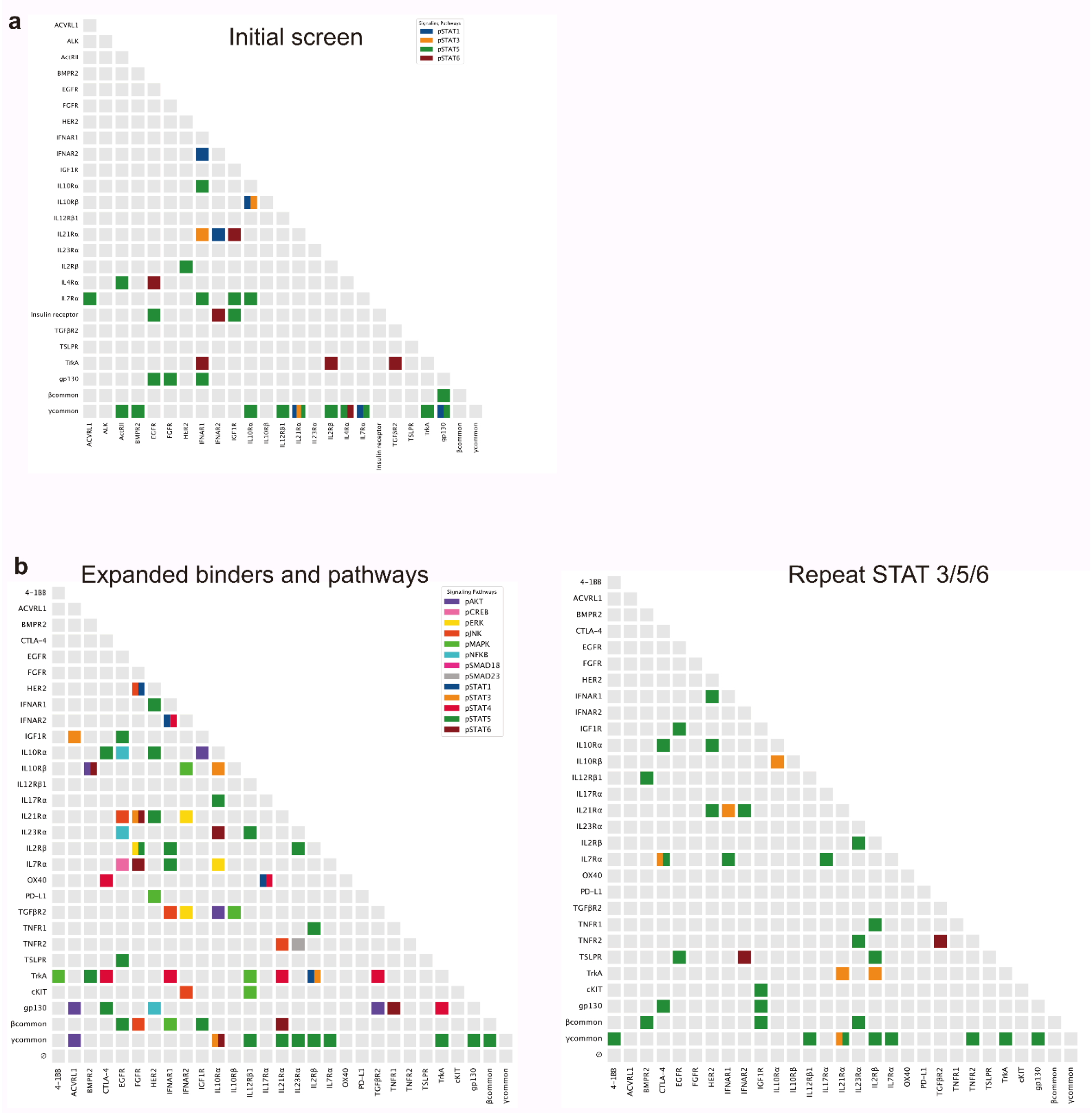

Supplementary Figure 2: All-by-all signaling activity from three experiments. (a) Initial results from signaling screen across the pSTAT1,3,5,6 pathways for the initial pilot of 576 fusions. (b) Results from signaling screen across 13 different signaling pathways and the full library of fusions. IL4R and Insulin receptor proteins are missing since they failed to transform in this run. (c) Repeating the experiment from (b) in an independent PBMC donor while assessing the pSTAT3,5,6 signaling pathways. pSTAT1 was attempted but the antibody batch used failed to capture positive controls and as a result was omitted.

bioRxiv preprint doi: https://doi.org/10.1101/2025.10.12.681920; this version posted October 13, 2025. The copyright holder for this preprint (which was not certified by peer review) is the author/funder, who has granted bioRxiv a license to display the preprint in perpetuity. It is made available under aCC-BY 4.0 International license.

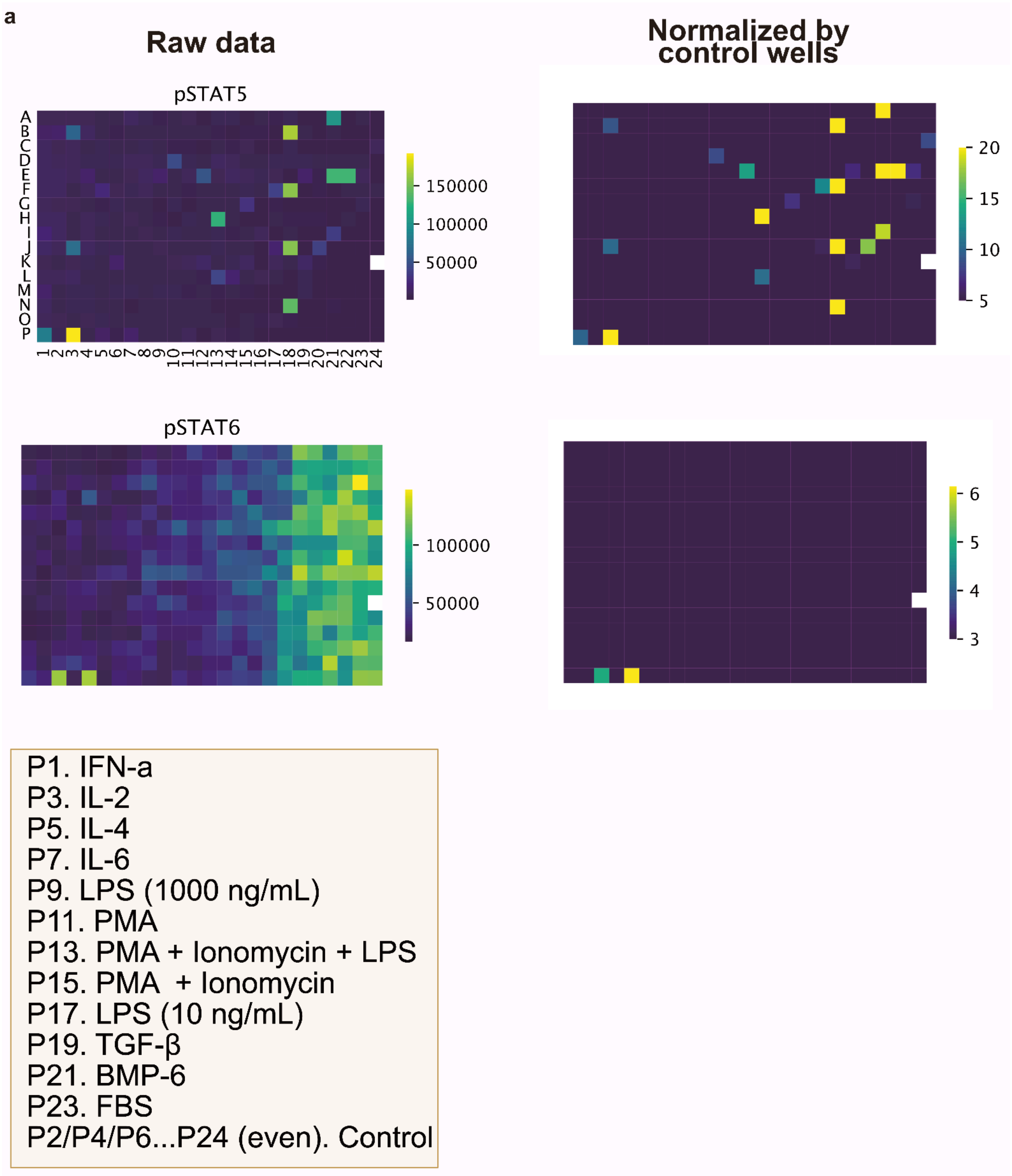

Supplementary Figure 3: Example of an all-by-all signaling activity plate measurement. (a) Signaling results from the all-by-all analysis across the STAT5,3,6 pathways for a subset of the data shown in Figure 3A. To control for batch effects, we included negative controls across each plate and pathway-specific positive controls to benchmark signaling responses.

bioRxiv preprint doi: https://doi.org/10.1101/2025.10.12.681920; this version posted October 13, 2025. The copyright holder for this preprint (which was not certified by peer review) is the author/funder, who has granted bioRxiv a license to display the preprint in perpetuity. It is made available under aCC-BY 4.0 International license.

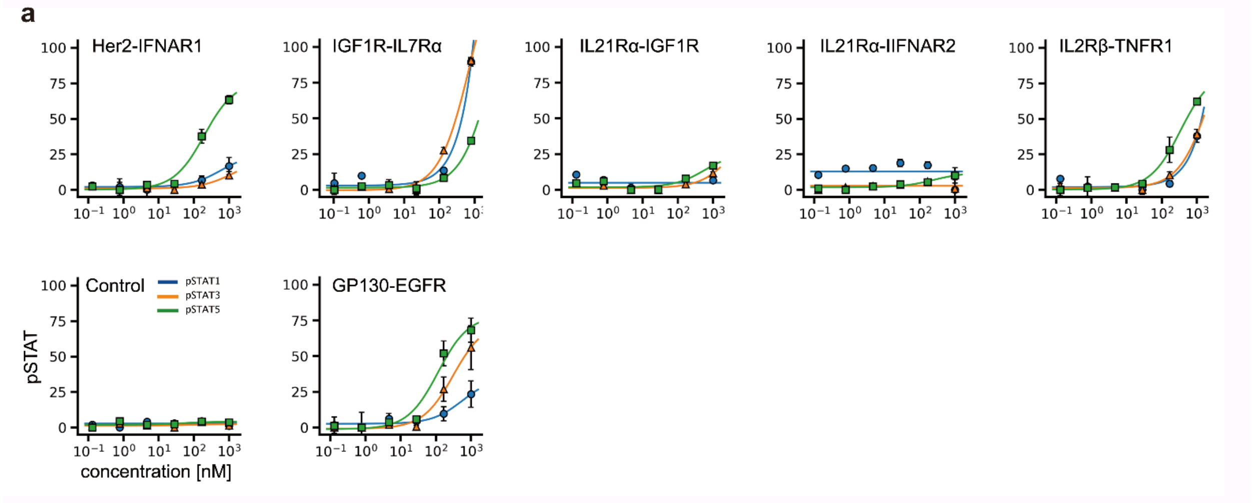

Supplementary Figure 4: Novokines that failed to pass further verifications. (a) Signaling results from the all-by-all analysis across the pSTAT1,3,5, 6 pathways for a subset of the seven hits that failed verifications test due to non saturating signals, or lack of signaling.

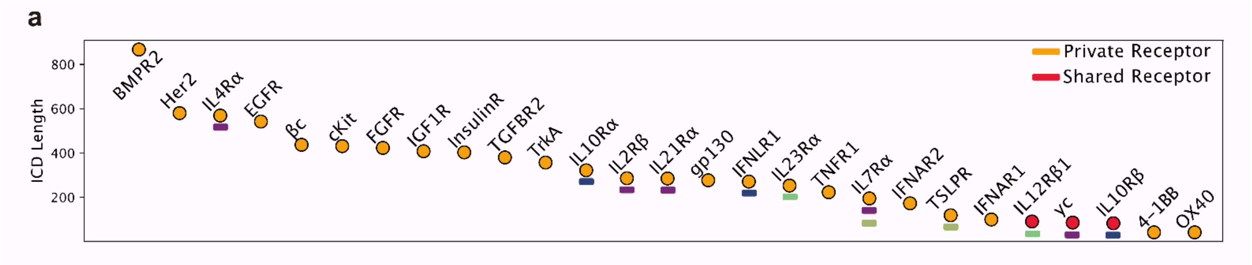

Supplementary Figure 5: ICD length across select receptors. (a) common receptors that form heteromeric signaling complexes are marked with a red circle. Lines under receptors match known pairs.

bioRxiv preprint doi: https://doi.org/10.1101/2025.10.12.681920; this version posted October 13, 2025. The copyright holder for this preprint (which was not certified by peer review) is the author/funder, who has granted bioRxiv a license to display the preprint in perpetuity. It is made available under aCC-BY 4.0 International license.

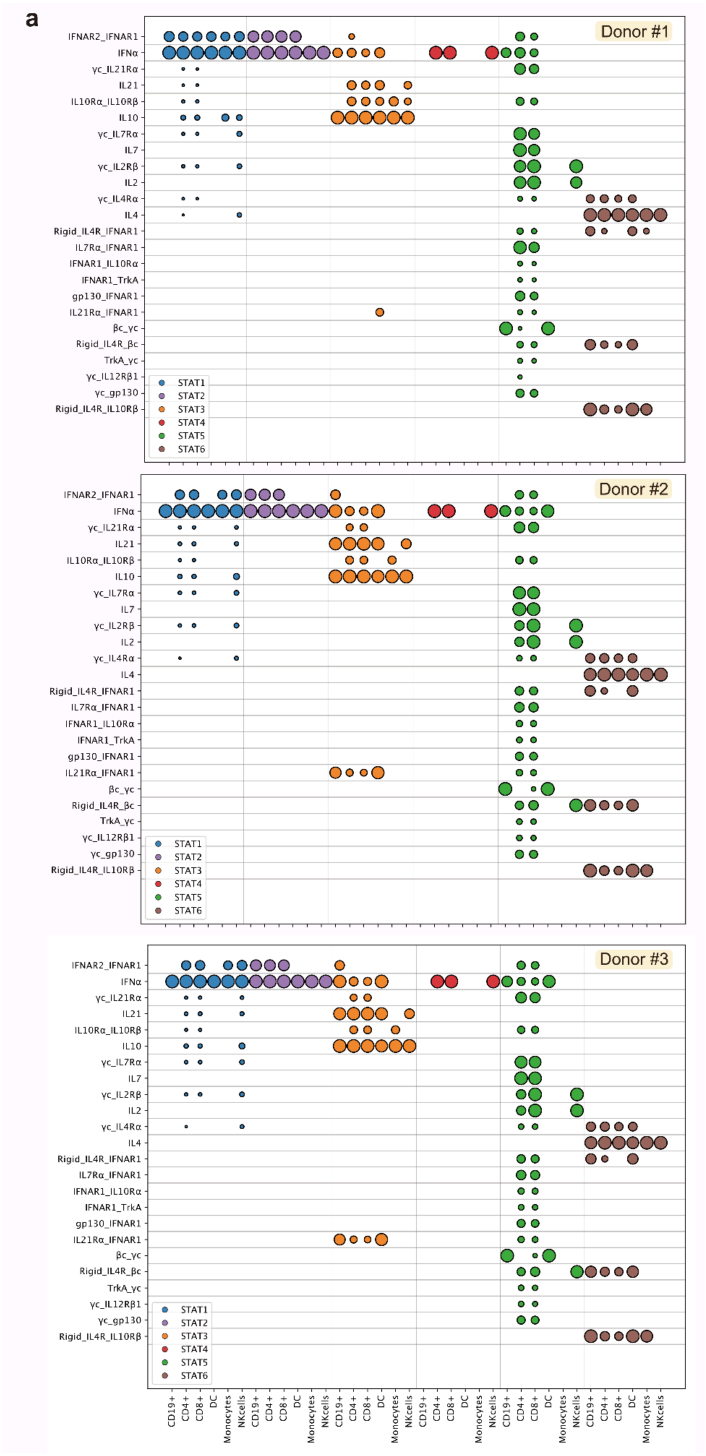

Supplementary Figure 6: Agonists activity across PBMC cell populations. (a) cell-specific activity of selected novel agonists in the pSTAT1, pSTAT2, pSTAT3, pSTAT4, pSTAT5 and pSTAT6 pathways across PBMC cell subtypes from the three donors tested.

bioRxiv preprint doi: https://doi.org/10.1101/2025.10.12.681920; this version posted October 13, 2025. The copyright holder for this preprint (which was not certified by peer review) is the author/funder, who has granted bioRxiv a license to display the preprint in perpetuity. It is made available under aCC-BY 4.0 International license.

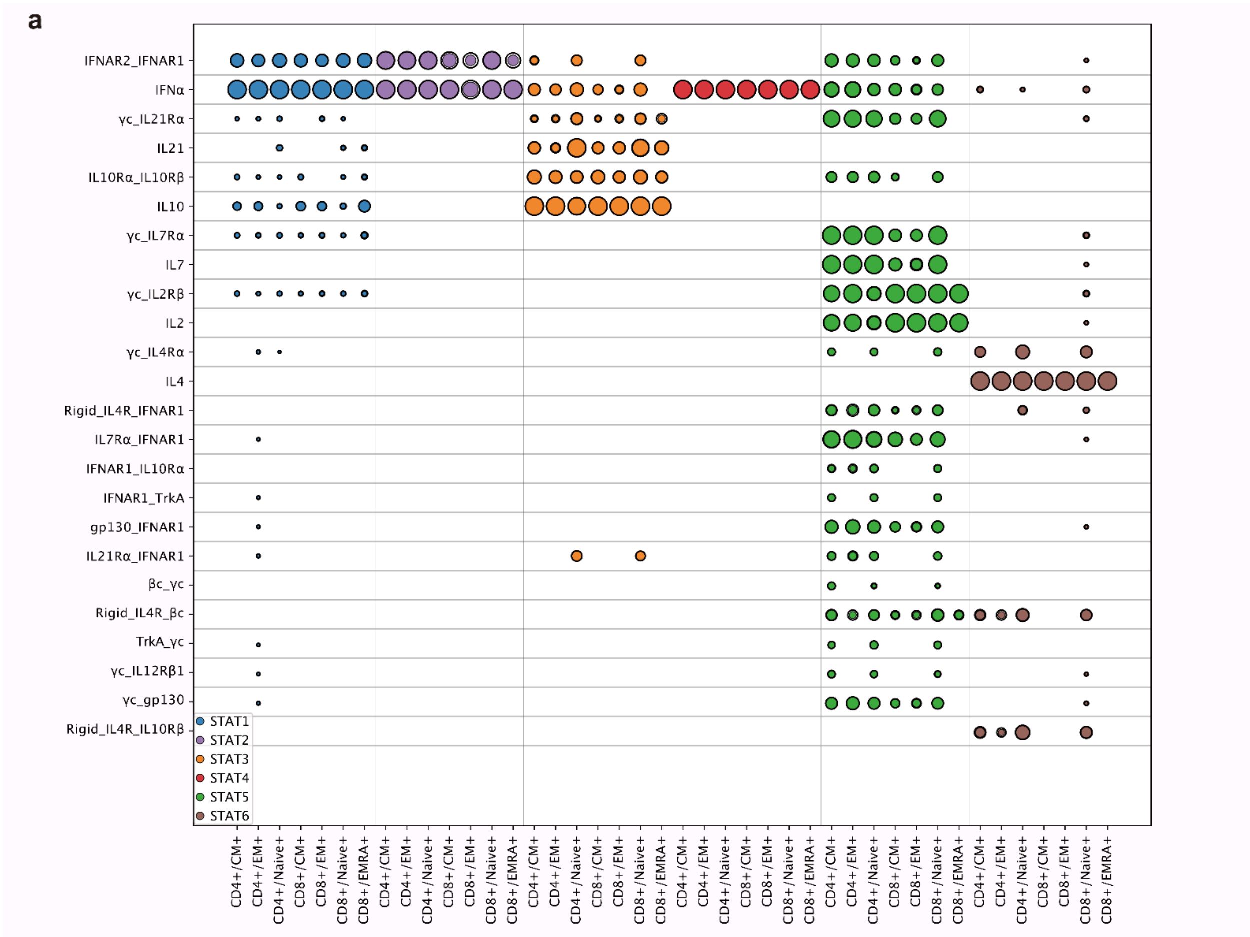

Supplementary Figure 7: Agonists activity across T-cell subsets. (a) cell-specific activity of selected novel agonists in the pSTAT1, pSTAT2, pSTAT3, pSTAT4, pSTAT5 and pSTAT6 pathways across T-cell subtypes. Results displayed are averages from the three donors tested.

bioRxiv preprint doi: https://doi.org/10.1101/2025.10.12.681920; this version posted October 13, 2025. The copyright holder for this preprint (which was not certified by peer review) is the author/funder, who has granted bioRxiv a license to display the preprint in perpetuity. It is made available under aCC-BY 4.0 International license.

Supplementary Table 1: List of receptor binding proteins generated in this study. (a) summary table detailing the receptor binding proteins generated in this study. The table includes information on the target receptor, signaling domain, affinity, size, and design method for each binder.

Receptor	Signaling domain	Affinity (nM)	Size (aa)	Method
γcommon	JAK3	2	56	Rosetta
IL2Rβ	JAK1	4	100	Agonist redesign
IL4Rα	JAK1	4	113	Agonist redesign
TSLPR	JAK2	10	64	Rosetta
IL10Rβ	TYK2	200	71	RF Diffusion
IL12Rβ1	TYK2	2	99	Rosetta concave
IFNAR1	TYK2	1	60	Rosetta
IFNAR2	JAK1	58	86	RF Diffusion
βcommon	JAK2	25	60	Rosetta

Supplementary Table 2: List of reagents used in this study.

Reagent	Catalog Number
BD OptiBuild BUV395 Rat Anti-Human CXCR5 (CD185)	740266
BUV496 Mouse Anti-Human CD3	612940
BUV563 Mouse Anti-Human CD56	612928
BUV661 Mouse Anti-Human HLA-DR	612981
BUV737 Mouse Anti-Human CD27	612829
BD OptiBuild BUV805 Mouse Anti-Human CD19	742007
Brilliant Violet 650 anti-human CD45RA Antibody	304136
BV750 Mouse Anti-Human CD4	566355
PerCP/Cyanine5.5 anti-STAT6 Phospho (Tyr641) Antibody	686010
BD Phosflow RB744 Mouse Anti-Stat5 (pY694)	570506
Phospho-Stat2 (Tyr690) (D3P2P) Rabbit mAb (PE Conjugate) #77366	77366S
BD Phosflow PE-CF594 Mouse Anti-Stat3 (pY705)	562673
BD Phosflow Alexa Fluor® 647 Mouse Anti-Stat1 (pY701)	562070
BD Phosflow R718 Mouse Anti-Stat4 (pY693)	567602
BD Pharmingen APC-H7 Mouse Anti-Human CD8	561423
Human TruStain FcX (Fc Receptor Blocking Solution)	422302
BD Phosflow Fix Buffer I	557870
BD Phosflow Perm Buffer III	558050
BD Cytofix™ Fixation Buffer	554655
BD Phosflow™ PE Mouse anti-Stat1 (pS727)	560069

bioRxiv preprint doi: https://doi.org/10.1101/2025.10.12.681920; this version posted October 13, 2025. The copyright holder for this preprint (which was not certified by peer review) is the author/funder, who has granted bioRxiv a license to display the preprint in perpetuity. It is made available under aCC-BY 4.0 International license.

BD Phosflow™ Alexa Fluor® 488 Mouse Anti-ERK1/2 (pT202/pY204)	612592
BD Phosflow™ Alexa Fluor® 647 Mouse Anti-Stat5 (pY694)	562076
BD Phosflow™ V450 Mouse Anti-Stat6 (pY641)	561203
BD Phosflow™ Alexa Fluor® 647 Mouse Anti-Stat1 (pY701)	562070
BD Phosflow™ Alexa Fluor® 488 Mouse Anti-Stat3 (pY705)	557814
BD Phosflow™ PE Mouse anti-Stat3 (pS727)	558557
BD Phosflow™ Pacific Blue™ Mouse anti-p38 MAPK (pT180/pY182)	560313
BD Phosflow™ Alexa Fluor® 488 Mouse Anti-Stat4 (pY693)	558136
BD Phosflow™ PE Mouse Anti-Akt (pT308)	558275
BD Phosflow™ BV421 Mouse Anti-Akt (pS473)	562599
BD Phosflow™ Alexa Fluor® 647 Mouse anti-JNK (pT183/pY185)	562481
BD Phosflow™ BV421 Mouse Anti-Human NF-κB p65 (pS529)	565446
BD Phosflow™ Alexa Fluor® 647 Anti-Smad2 (pS465/pS467)/Smad3 (pS423/pS425)	562696
BD Phosflow™ Alexa Fluor® 488 Mouse Anti-CREB (pS133) / ATF-1 (pS63)	558435
BD Phosflow™ PE Rat anti-Smad1 (pS463/pS465)/Smad8 (pS465/pS467)	562509
EasySep™ Human Naïve Pan T Cell Isolation Kit	17961
EasySep™ Human CD8+ T Cell Isolation Kit	17953
EasySep™ Human CD4+ T Cell Isolation Kit	17952
Dynabeads™ Human T-Activator CD3/CD28 for T Cell Expansion and Activation	11131D
Human IL-7, research grade	130-095-367
Human IL2-IS, research grade	130-097-743
Human IL-4	130-093-917
Human IL-10, research grade	130-093-948
Recombinant Human IL-21 Protein	8879-IL-050/CF
Interferon alpha 1/IFNA1 Protein, Human, Recombinant (His Tag)	12341-H08Y
BD OptEIA™ Human IFN-γ ELISA Set	555142
The BD OptEIA™ Reagent Set B	550534
